# Prevalence of symptoms, comorbidities, and reinfections in individuals infected with Wild-Type SARS-CoV-2, Delta, or Omicron variants: a comparative study in western Mexico

**DOI:** 10.3389/fpubh.2023.1149795

**Published:** 2023-04-27

**Authors:** Marcela Peña Rodríguez, Jorge Hernández Bello, Natali Vega Magaña, Oliver Viera Segura, Mariel García Chagollán, Hazael Ramiro Ceja Gálvez, Jesús Carlos Mora Mora, Francisco Israel Rentería Flores, Octavio Patricio García González, José Francisco Muñoz Valle

**Affiliations:** ^1^Laboratorio de Enfermedades Emergentes y Reemergentes, Centro Universitario de Ciencias de la Salud, Universidad de Guadalajara, Guadalajara, Mexico; ^2^Instituto de Investigación en Ciencias Biomédicas, Centro Universitario de Ciencias de la Salud, Universidad de Guadalajara, Guadalajara, Mexico; ^3^Doctorado en Ciencias en Biología Molecular en Medicina, Centro Universitario de Ciencias de la Salud, Universidad de Guadalajara, Guadalajara, Mexico; ^4^Centro Universitario de Ciencias de la Salud, Universidad de Guadalajara, Guadalajara, Mexico; ^5^Translational Institute of Genomic Singularity (ITRASIG), Irapuato, Mexico

**Keywords:** COVID-19, variants, Delta, Omicron, SARS-CoV-2

## Abstract

**Introduction:**

The variants of severe acute respiratory syndrome coronavirus 2 (SARS-CoV-2) have been classified into variants of interest (VOIs) or concern (VOCs) to prioritize global monitoring and research on variants with potential risks to public health. The SARS-CoV-2 high-rate mutation can directly impact the clinical disease progression, epidemiological behavior, immune evasion, vaccine efficacy, and transmission rates. Therefore, epidemiological surveillance is crucial for controlling the COVID-19 pandemic. In the present study, we aimed to describe the prevalence of wild-type (WT) SARS-CoV-2 and Delta and Omicron variants in Jalisco State, Mexico, from 2021 to 2022, and evaluate the possible association of these variants with clinical manifestations of COVID-19.

**Methods:**

Four thousand and ninety-eight patients diagnosed with COVID-19 by real-time PCR (COVIFLU, Genes2Life, Mexico) from nasopharyngeal samples from January 2021 to January 2022 were included. Variant identification was performed by the RT-qPCR Master Mut Kit (Genes2Life, Mexico). A study population follow-up was performed to identify patients who had experienced reinfection after being vaccinated.

**Results and Discussion:**

Samples were grouped into variants according to the identified mutations: 46.3% were Omicron, 27.9% were Delta, and 25.8% were WT. The proportions of dry cough, fatigue, headache, muscle pain, conjunctivitis, fast breathing, diarrhea, anosmia, and dysgeusia were significantly different among the abovementioned groups (*p* < 0.001). Anosmia and dysgeusia were mainly found in WT-infected patients, while rhinorrhea and sore throat were more prevalent in patients infected with the Omicron variant. For the reinfection follow-up, 836 patients answered, from which 85 cases of reinfection were identified (9.6%); Omicron was the VOC that caused all reported reinfection cases. In this study, we demonstrate that the Omicron variant caused the biggest outbreak in Jalisco during the pandemic from late December 2021 to mid-February 2022 but with a less severe form than the one demonstrated by Delta and WT. The co-analysis of mutations and clinical outcomes is a public health strategy with the potential to infer mutations or variants that could increase disease severity and even be an indicator of long-term sequelae of COVID-19.

## 1. Introduction

Severe acute respiratory syndrome coronavirus 2 (SARS-CoV-2) is a positive-sense, single-stranded RNA beta-coronavirus responsible for coronavirus disease 2019 (COVID-19) (1). According to the Johns Hopkins Coronavirus Resource Center, until November 23, 2022, 639,511,643 cases have been reported worldwide, and 7,125,176 cases have been reported in México. Although the SARS-CoV-2 encodes a 3′–5′-exoribonuclease that permits high-fidelity replication by the viral RNA-dependent RNA polymerase (RdRP), numerous viral genome mutations have been reported, from which viral variants have originated (2).

Multiple studies have demonstrated that mutations, especially in the spike (S) gene of SAR-CoV-2, can directly impact the clinical disease progression, epidemiological behavior, immune evasion, vaccine efficacy, and transmission rate, attributed to the crucial role of this protein in the host cell’s viral entry by binding with the host cell receptor angiotensin-converting enzyme-2 (ACE2) (3–5). Therefore, mutations in the S gene have been extensively studied.

In late 2020, the World Health Organization (WHO) exhorted virus genomic surveillance to detect “signals” of potential variants of interest (VOIs) and assess these based on the risk posed to global public health (6). Hence, the WHO prompted the characterization and classification of VOIs and variants of concern (VOCs) to prioritize global monitoring and research on variants with potential risks to public health. This classification is based on their genome mutations, higher spreading properties, association with disease severity, and immune response evasion (6).

The Delta and Omicron VOCs, including the B.1.617.2 and AY and the B.1.1.529 and BA lineages, respectively, have caused the most extensive outbreaks of COVID-19 compared to the wild-type (WT) virus (7).

At a molecular level, key virus mutations located in the S protein receptor-binding domain (RBD) are critical for the antibody resistance and infectivity of the SARS-CoV2 variant (8). In this sense, the Delta variant generated the most hazardous and widespread effects (9). Specifically, some reported mutations that had a more significant biological impact in this VOC were L452R and T478K, both of which are also present in the Omicron VOC (10). On the other hand, some of Omicron’s representative mutations in the RBD are Del H69 -, K417N, E484A, and N501Y (8, 11).

The Omicron variant has mutations described in other VOCs and has yielded at least six genetically related viral sublineages (BA.1, BA.1.15, BA.2, BA.2.12.1, BA.4, and BA.5). This variant has a higher potential for transmission than the Delta variant, which is mediated via its higher ACE2 affinity and the high number of mutations in the SARS-CoV-2 complete genome (12, 13). Some studies have shown that the Omicron variant has a lower replication rate in lung cells than the B.1.617.2 (Delta) (14). Nevertheless, Omicron is mainly characterized by its ability to evade the humoral immune response in fully vaccinated individuals (including the booster dose); therefore, Omicron is considered more infectious (2.7–3.7 times higher) than the Delta variant (12, 15, 16).

Even though there is extensive research on the impact of the virus mutation in its transmission and receptor affinity, information regarding the association of specific symptoms with a particular VOC is meager. Nevertheless, some mutations present in VOCs, such as the mutation S194L found in the Nucleocapsid gene, have been associated with symptomatic patients (17) or a higher frequency of fatal outcomes (18). This could indicate the crucial role of some variants in elevating the pathogenicity of the virus, thereby contributing to increased symptoms of disease severity (8).

Based on the above, the genomic surveillance of SARS-CoV-2 during disease outbreaks is imperative. It effectively identifies the appearance of mutations that change the virus phenotype and enables the tracking of viral evolution. Whole-genome and amplicon-based sequencing are the preferred techniques to characterize viruses genetically (19). Nevertheless, this approach can result in expensive, slow, and complex processes because of the highly qualified personnel required for its fulfillment. In addition, the economic gap has made this difficult for developing countries such as Mexico to rely on these technologies entirely; therefore, other validated quantification methods for variant detection, such as screening SNP assays via RT-qPCR technology, have been extensively employed (20, 21).

In this context, this study aimed to identify SARS-CoV-2 variants via a SARS-CoV-2 VOC PCR screening test and determine their association with clinical manifestations of the disease to support the relevance of variants in a clinical setting.

## 2. Materials and methods

### 2.1. Study population

As part of the strategies of the Health Situation Room of the University of Guadalajara (22) to address the COVID-19 pandemic in the State of Jalisco, Mexico, a university system of molecular epidemiological surveillance was created. In this context, 4,092 patients diagnosed with COVID-19 from January 2021 to January 2022 were included. The COVID-19 diagnosis was made by real-time PCR (COVIFLU, Genes2Life, Mexico) using nasopharyngeal samples in the Laboratorio de Diagnóstico de Enfermedades Emergentes y Reemergentes (LaDEER), Guadalajara, Jalisco, Mexico. The RT-qPCR processing and sample collection details were previously described elsewhere (23).

Clinical and epidemiological information from these patients was obtained during diagnosis in a database containing personal information, comorbidities, symptoms, travel history, and previous contact with positive COVID-19 cases. Furthermore, as a part of a follow-up to evaluate the clinical outcomes of SARS-CoV-2 reinfections cases from February to March 2022, all participants were contacted by telephone call 2–6 months after the diagnosis.

### 2.2. Variant identification

Following diagnosis, positive SARS-CoV-2 samples with a Ct-value of <27 of the N2 region gene were processed by the RT-qPCR Master Mut Kit (Genes2Life, Mexico), which has been validated as a rapid SARS-CoV-2 VOC screening test (20). This assay was performed according to the manufacturer’s instructions. Furthermore, 63 randomly selected samples were sequenced to validate the results further.

### 2.3. Statistical analysis

Tables were made with SPSS (IBM SPSS, Statistics, Chicago, IL, United States). Categorical (qualitative) variables were summarized as frequencies and percentages. For the comparison of proportions among groups, the chi-square test was employed. Quantitative variables are presented as mean ± standard deviation; ANOVA was carried out with the Bonferroni multiple comparison test for comparative analyses. A *p*-value of *p* < 0.05 was considered statistically significant. The pie chart and Sankey plot were compiled using GraphPad Prism v9.1 (Graph-Pad Company, San Diego, CA, United States) and R v4.1.2 (R core Team, Vienna, Austria), respectively.

## 3. Results

### 3.1. Clinical, demographic, and epidemiological characteristics of COVID-19 patients

**Figure 1:**
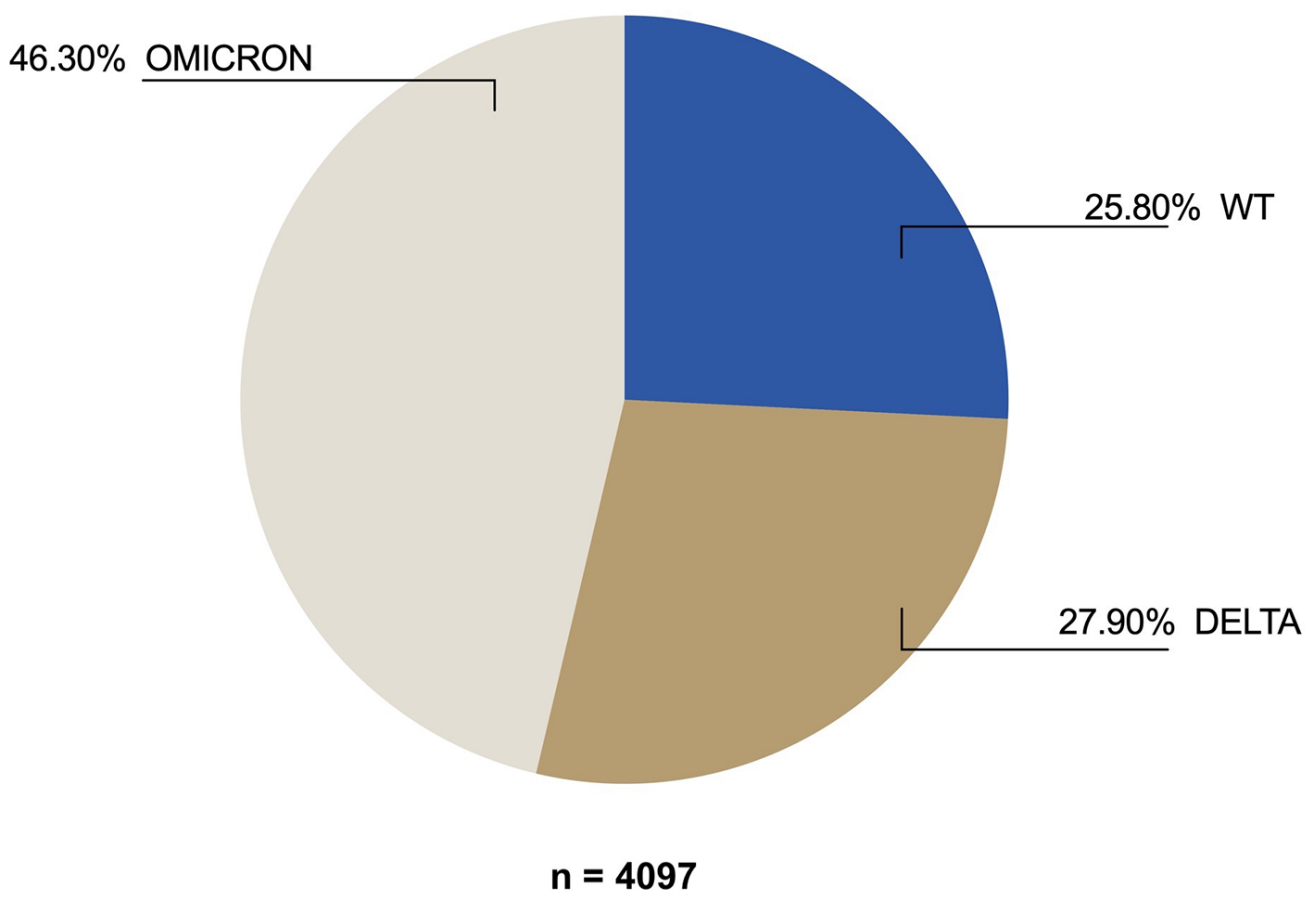
VOC frequency among SARS-CoV-2-positive patients diagnosed from January 2021 to January 2022.

A total of 4,097 samples of COVID-19 patients were analyzed and grouped into variants according to the identified mutations: 46.3% were Omicron, 27.9% were Delta, and 25.8% were WT (Figure 1). Another group (*n* = 85) consisted of various variants identified as Alpha, Beta, Gamma, and Kappa; however, this was not considered in the samples analyzed (*n* = 4,097) for downstream analysis since the number of samples in that group was scarce.

The demographic and underlying disease information of the studied population is shown in Table 1. The age was different between individuals infected with different SARS-CoV-2 variants (WT, Delta, or Omicron); in particular, the individuals infected with the Delta variant presented a lower mean age than those infected with Omicron or WT (*p* = 0.004 and *p* < 0.001, respectively).

**Table 1:** Clinical, demographic, and epidemiological characteristics of COVID-19 patients diagnosed in Jalisco, Mexico, from January 2021 to January 2022.

	Omicron *n* = 1,898	Delta *n* = 1,142	WT *n* = 1,057	*p*-value
Age (years), mean SD±	38.76 ± 15.27	36.74 ± 16.91	40.62 ± 15.16	<0.001 φ 0.004 ϯ
Age groups (years)				
0–19 years	153 (39.0)	180 (45.9)	59 (15.0)	<0.05 Ϯ, ψ, φ
20–39 years	891 (47.4)	515 (27.4)	473 (25.2)	<0.05 Ϯ, ψ
40–59 years	657 (48.2)	303 (22.2)	403 (29.6)	<0.05 Ϯ, φ
>60 years	195 (42.3)	143 (31.0)	123 (26.7)	NS
Gender				
Female ^n (%)^	1,116 (58.8)	647 (56.7)	594 (56.1)	0.12
Male ^n (%)^	765 (40.3)	490 (42.9)	460 (43.5)	0.12
Underlying diseases (comorbidities)				
Obesity ^n (%)^	225 (11.9%)	182 (15.9)	248 (23.4)	<0.001ϯ, ψ,φ
Smoking ^n (%)^	194 (10.2)	183 (16.0)	169 (16.0)	<0.001 ϯ, ψ
Arterial hypertension ^n (%)^	139 (7.3)	114 (10.0)	156 (14.7)	<0.001 ϯ, ψ, φ
Diabetes ^n (%)^	100 (5.3)	76 (6.7)	98 (9.3)	<0.001 ψ
Asthma ^n (%)^	57 (3.0)	51 (4.5)	49 (4.6)	0.03 NS
Travel history ^n (%)^	124 (6.5)	109 (9.5)	129 (12.2)	<0.001 ϯ, ψ

Comparison by groups: ϯ Omicron vs. Delta; ψ Omicron vs. WT; φ Delta vs. WT.

NS, Not significant (*p* > 0.05). The chi-square test and ANOVA were employed for statistical analysis.

There were no differences found among the sex proportions of the studied groups. All study groups’ five most prevalent comorbidities and risk factors were obesity, smoking, arterial hypertension, diabetes, and asthma (Table 1). Obesity prevalence differed among the three groups; generally, patients infected with WT had a higher prevalence of obesity than the other groups (<0.001). Regarding smoking, patients infected with Delta or WT had a higher prevalence of this variable than those infected with Omicron (<0.001). On the other hand, arterial hypertension was more prevalent in patients infected with WT than those infected with Delta or Omicron. Diabetes only differed between patients infected with WT vs. Omicron; this comorbidity was higher in the WT group (*p* < 0.001). No difference was found in asthma prevalence among infected patients with different variants (Table 1).

The travel history of previous infections was significantly less present in the Omicron group than in Delta-and WT-positive patients (Table 1).

### 3.2. Genomic SARS-CoV-2 diversity during the pandemic in Jalisco state

**Figure 2:**
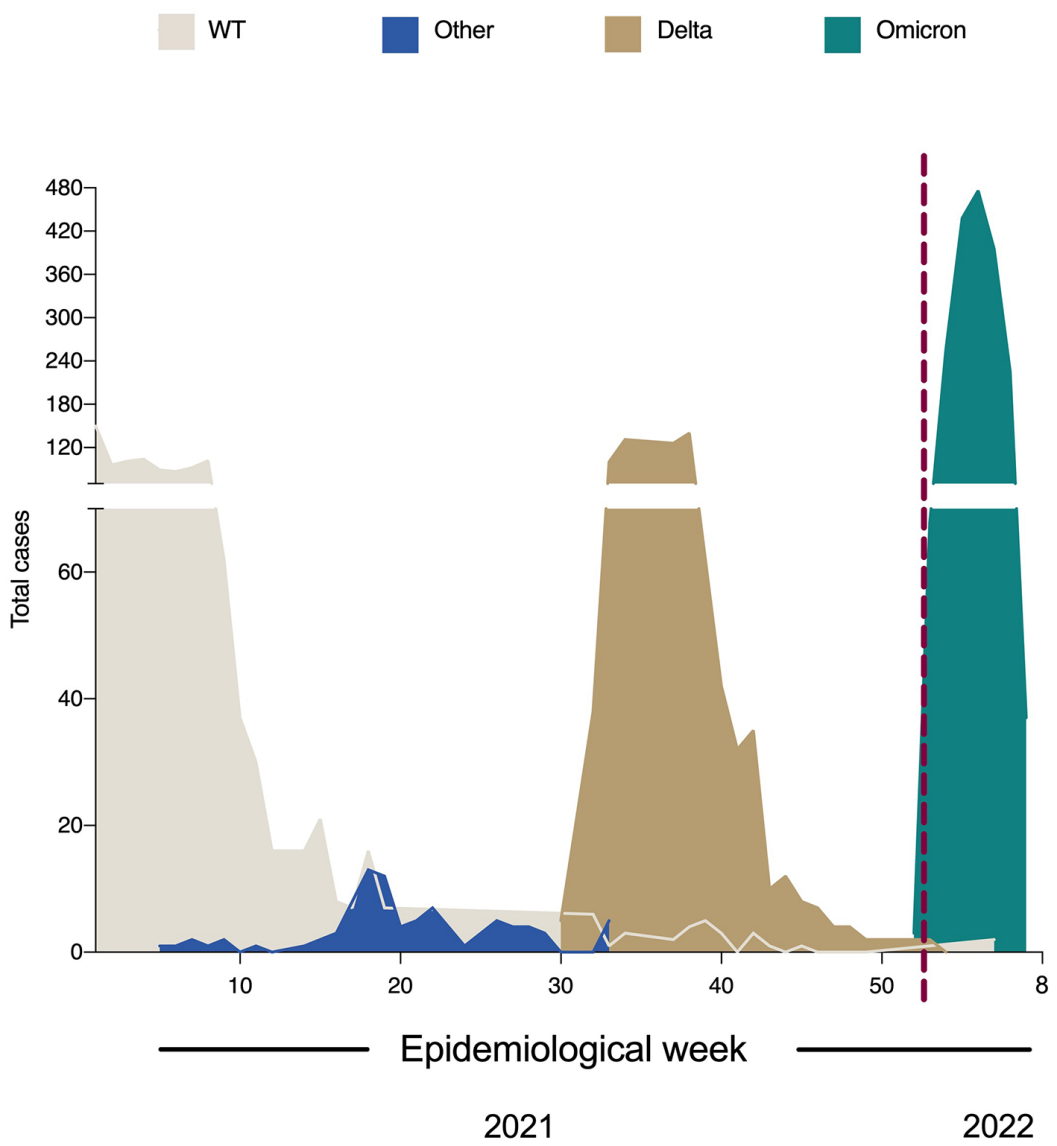
Change in the prevalence of SARS-CoV-2 variants in Jalisco, Mexico (January 2021–January 2022). The total number of cases was 4,097; namely, the total cases of WT, other, Delta, and Omicron variants were 1,057, 85, 1,142, and 1898, respectively.

According to our analysis, mainly WT-like variants circulated at the beginning of 2021; however, they showed a progressive replacement pattern by other variants, which became dominant over WT over the weeks. From the 30th epidemiological week, B.1.617.2 (Delta) switched the genome pattern observed to date and established itself as the dominant variant in the area. A similar phenomenon was registered in the region in 2022, in which BA (Omicron) lineages substituted the predominance of Delta (Figure 2).

### 3.3. Difference in the frequency of COVID-19 symptoms according to VOC

We compared the most reported symptoms among the Omicron and Delta VOC and WT-infected patients. Most symptoms significantly differed among the three studied groups. Specifically, the proportions of dry cough, fatigue, headache, muscle pain, conjunctivitis, fast breathing, diarrhea, anosmia, and dysgeusia were significantly different among the abovementioned groups (*p* < 0.001).

Anosmia and dysgeusia were mainly found in WT-infected patients, while rhinorrhea and sore throat were more prevalent in patients infected with the Omicron variant.

When comparing the Omicron group, fever and nasal congestion were significantly less prevalent concerning the WT and Delta variant (*p* < 0.001); furthermore, the frequency of having difficulty breathing was lower in the Omicron group than in the WT (*p* < 0.001; Table 2).

**Table 2:** Frequency of COVID-19 symptoms in patients infected with SARS-CoV-2 variants of concern (VOC).

	Omicron *n* = 1,898 n (%)	Delta *n* = 1,142 n (%)	WT *n* = 1,057 n (%)	*p*-value
Symptoms				
Asymptomatic	446 (23.5)	46 (4.0)	13 (1.2)	<0.001 ϯ, ψ, φ
Fever	699(36.8)	569 (49.8)	517 (48.9)	<0.001 ϯ, ψ
Dry cough	905 (47.7)	660 (57.8)	679 (64.2)	<0.001 ϯ, ψ, φ
Nasal congestion	388 (20.4)	334 (29.2)	331 (31.3)	<0.001 ϯ, ψ
Chest pain	260 (13.7)	168 (14.7)	222 (21.0)	<0.001 ψ, φ
Fatigue	527 (27.8)	473 (41.4)	537 (50.8)	<0.001 ϯ, ψ, φ
Productive cough	262 (13.8)	196 (17.2)	148 (14.0)	0.02 ϯ
Difficulty breathing	154 (8.1)	130 (11.4)	144 (13.6)	<0.001 ϯ, ψ
Headache	1,004 (52.9)	743 (65.1)	784 (74.1)	<0.001 ϯ, ψ, φ
Muscle pain	507 (26.7)	421 (36.9)	453 (42.8)	<0.001 ϯ, ψ, φ
Rhinorrhea	582 (30.7)	333 (29.2)	300 (28.4)	0.3 NS
Sore throat	928 (48.9)	503 (44.0)	526 (49.7)	0.01 ϯ, φ
Conjunctivitis	213 (11.2)	235 (20.6)	332 (31.4)	<0.001 ϯ, ψ, φ
Diarrhea	99 (5.2)	155 (13.6)	203 (19.2)	<0.001 ϯ, ψ, φ
Fast breathing	7 (0.4)	31 (2.7)	56 (5.3)	<0.001 ϯ, ψ, φ
Anosmia	149 (7.9)	425 (37.2)	476 (45.0)	<0.001 ϯ, ψ, φ
Dysgeusia	163 (8.6)	402 (35.2)	426 (40.3)	<0.001 ϯ, ψ, φ

Comparison by groups: ϯ Omicron vs. Delta; ψ, Omicron vs. WT; φ, Delta vs. WT. Wild-type = WT.

The chi-square test was employed for statistical analysis.

The asymptomatic population was more prevalent in the Omicron group than the Delta and WT groups (23.3% vs. 4.4% vs. 1.9%; *p* < 0.001, respectively). Most symptoms were less frequent in the Omicron group than in the Delta and WT groups, except for sore throat and rhinorrhea. The sore throat was less frequent in the Delta group than in WT and Omicron patients. In contrast, rhinorrhea was more present in the Omicron group than the others; however, this was not significant (*p* = 0.06; Table 2).

**Figure 3:**
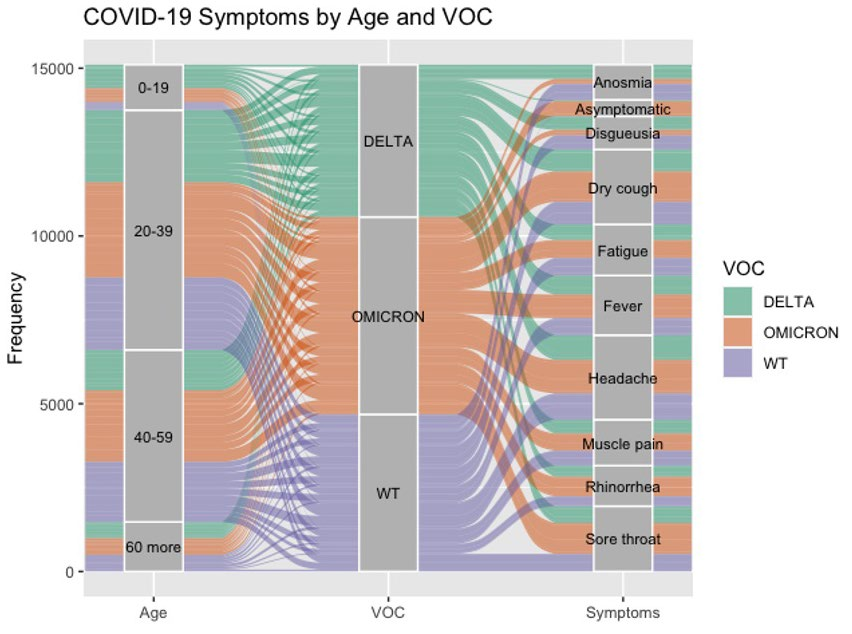
Interrelationship of COVID-19, age, VOC, and symptoms. The color indicates the VOC: WT is represented in light purple, Delta is represented in green, and Omicron is represented in orange. The pathway’s size (line) shows the related quantities between the compared sets. Results are shown in the number of cases obtained (frequency).

For the visual identification of the interrelationship of COVID-19 variants with symptoms, a Sankey plot stratified by age was constructed (Figure 3).

### 3.4. Number of symptoms and comorbidities among SARS-CoV-2 patients infected with different VOCs

Since the beginning of the pandemic, Jalisco state promoted community containment, and social distancing once the state reactivated its activities. In this context, the state coordinated efforts with the University to encourage prompt SARS-CoV-2 testing of the population by having free antigen and PCR tests for the community. Therefore, symptomatic, or asymptomatic people at risk of contact with an infected individual came to the laboratory for a free SARS-CoV-2 test; this happened during the different waves of the pandemic.

Therefore, symptoms were divided into the following categories: asymptomatic, 1–3, 4–6, and more than seven reported symptoms (Table 3). Patients infected with Omicron were the most asymptomatic group (23.5%) compared to those infected with Delta or WT (4 and 1.2%, respectively). On the other hand, individuals infected with the WT virus reported more symptoms (>7) than individuals infected with the Omicron and Delta variants (53.8% vs. 28 and 4.8%, respectively, *p* < 0.001).

**Table 3:** Number of symptoms and comorbidities among COVID-19 patients infected with different SARS-CoV-2 VOCs.

Symptoms	Total *n* = 4,097 n (%)	Omicron *n* = 1,898 n (%)	Delta *n* = 1,142 n (%)	WT *n* = 1,057 n (%)	*p*-value
Asymptomatic	505 (12.3)	446 (23.5)	46 (4.0)	13 (1.2)	<0.001 ϯ, ψ, φ
1–3	436 (10.6)	234 (12.3)	140 (12.3)	62 (5.9)	<0.001 ψ, φ
4–6	1,557 (38.0)	687 (36.2)	456 (39.9)	414 (39.1)	NS
>7	1,600 (39.0)	531 (28.0)	500 (4.8)	569 (53.8)	<0.001 ϯ, ψ, φ
Comorbidities					
0	2,556 (62.4)	1,351 (71.2)	690 (60.4)	515 (48.7)	<0.001 ϯ, ψ, φ
1–3	1,529 (37.3)	541 (28.5)	449 (39.3)	539 (50.9)	<0.001 ϯ, ψ, φ
>4	13 (0.3)	6 (0.3)	3 (0.3)	4 (0.4)	NS

Comparison by groups: ϯ Omicron vs. Delta; ψ Omicron vs. WT; φ Delta vs. WT. WT, Wild-type.

The Chi-square test was employed for statistical analysis.

Regarding comorbidities, the WT group reported the most significant proportion of patients with 1–3 comorbidities, while the Omicron group had the highest proportion of patients without comorbidity. Patients with more than four comorbidities were the same among the three groups.

A study population follow-up was performed to identify patients who experienced reinfection after being vaccinated; 836 patients answered, from which 85 cases of reinfection were identified (9.6%). Omicron was the VOC that caused all reported reinfection cases. We did not observe another consistent clinical–demographic pattern among those reinfected patients, including age, comorbidities, or gender (data not shown).

**Figure 4:**
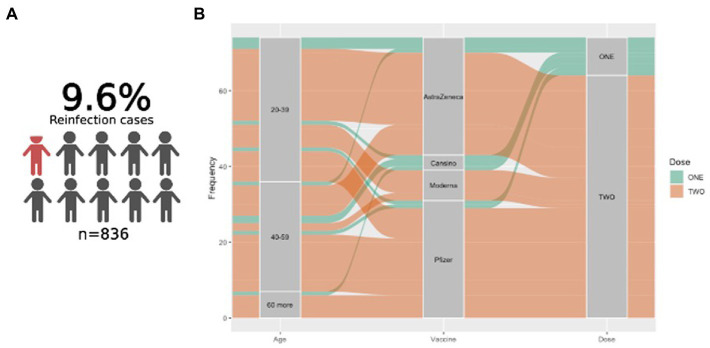
Reinfection cases in the studied population. **(A)** Graphical representation of patients who experienced reinfection after vaccination. **(B)** Sankey plot showing the reinfected cases according to age, vaccine platform, and vaccine received.

To visualize the behavior of reinfection cases, a Sankey diagram was constructed according to age, vaccine, and dose number received. In this manner, most reinfection cases identified reported a complete vaccination (2 doses) with AstraZeneca and Pfizer platforms (Figures 4A,B).

## 4. Discussion

The deployment of genomic surveillance to determine the geographic dissemination of SARS-CoV-2 variants has proven to be a valuable tool that has guided public health institutions in the decision-making process at local, regional, or even national levels, identifying the variants’ emergence in 2021 in a timely manner. Since the emergence of B.1.1.7 lineage in September 2020 and the infection cases increased in early 2021, many other variants have been identified (24).

In Mexico, Jalisco was the first State to confirm the first virus variant (namely Zeta variant) in February 2021, also known as the P.2 lineage (25); after that, other known variants appeared in the region. In the present study, we aim to describe the prevalence of SARS-CoV-2 variants in this Mexican State from 2021 to 2022 and evaluate the possible association of these variants with clinical manifestations of COVID-19.

Overall, in Jalisco state, 1,096 SARS-CoV-2 complete genome sequences from 2020 to May 01, 2021, were reported on the GISAID platform, a period in which the AY lineage (Delta variant) was prevalent. After that period, we identified Omicron as the VOC with more infection cases using the SNP screening RT-PCR method.

A total of 86,802 complete genome sequences in Mexico were reported from March 2020 to April 04, 2021, on the Mexican Consortium of Genomic Surveillance (MCGS) platform. Data published in this surveillance platform demonstrated that five lineages dominated the pandemic period of 2019–2022, and they were distributed over five waves. In the first wave, April 2020, B.1, B.1.1, and B.1.1.222 predominated over 2019 and 2020, reporting a change in the epidemiological pattern from 2021 onwards. At the beginning of 2021, B.1.1.519 replaced previous lineages as the prevalent variant in the country over the first three epidemic waves. Meanwhile, AY and BA lineages dominated the country’s fourth and fifth epidemic waves, completely substituting previously reported lineages (26). In Jalisco state, a similar behavior was reported; however, the third wave was characterized by a short period of the predominance of the P.2 lineage and B.1.1.519 at the beginning of 2021. As reported nationally, BA and AY lineages substituted the previous SARS-CoV-2 variant on the fourth and the fifth epidemic wave (26). Our discriminate mutation system for lineage classification identified the observed pattern described by the MCGS without requiring complete genome sequencing, shortening the reporting and analysis times for a quick decision-making process.

According to Jalisco’s total GISAID complete genome sequences during the COVID-19 pandemic, the first variant officially reported was P.2; however, the P.1 variant was the earliest VOI that was retrospectively isolated in Jalisco (17, 24). The emergence of P.1 and P.2 variants in the State matches the national pattern described, where a high number of COVID-19 cases caused by these variants was reported in epidemiological weeks one to five from 2021 (27). The augmented case rate in the weeks with more viral dispersion was associated with the high national and international mobility reported by the national council of transport and communication, augmented during December 2020 and January 2021 (28). Following the global spread of May 2021, Mexico began to register VOC-Delta in the second semester of the year, which became predominant during the rest of 2021 (29). Thereof, we reported that sublineages AY.26, AY.20, and AY.3 were genome sequences that were mostly reported in GISAID; this is consistent with the previous national description in which AY.26 and AY.20 were also the most prevalent (29), corroborating a homogeneous viral dispersion and providing information that national health councils could exploit.

According to the WHO, the VOCs that are currently circulating are the Delta (B.1.617.2) and Omicron (B.1.1.529) variants. For instance, the identification of Omicron (B.1.1.529) as a variant of concern on November 26, 2021, in South Africa revealed a new threat (30), as it was responsible for the fourth and last reported SARS-CoV-2 wave (31). Although contrasting the Delta variant, the Omicron variant was more rapidly spread; it was officially first detected in Mexico on December 03, 2021, and on January 05, 2022, in Jalisco state; however, the earliest case in the State was observed on December 29, 2021.

The spreading capacity can measure the impact of genome mutations on the epidemiological pattern; this behavior was observed in this study by comparing Omicron, Delta, and WT frequencies from January 2020 to January 2021, where Omicron not only exceeded the total number of cases previously reported but also displaced the Delta VOC. It is essential to consider that most specific amino acid substitutions that alter the viral transmission, severity, and neutralizing antibody evasion are located in the viruses’ S protein as it is one of the mutational hot spots (32).

The increased transmissibility of Omicron could be multifactorial and derived from the viral load, respiratory symptoms, viral shedding, mobility, previous contact, age, and vaccination status, among others (33, 34). Nevertheless, as to the viral load, it has been reported that it might be unrelated to higher transmissibility, as described previously for other variants (33). We observed lower travel history and previous contact with symptomatic patients among Omicron cases compared with Delta and WT, which could reflect the variant’s spread capacity, even in transmission from presymptomatic cases. This could be related to an unprecedented number of mutations in the Omicron variant, especially in the S protein (at least 35), which can influence its transmissibility (35). Specifically, the N501Y mutation has been reported in the Omicron and Alpha VOC as a decisive substitution that increases their transmissibility due to its increased affinity with the ACE2 receptor (36, 37), along with S477N, T478K, Q493R, Q496S, and Q498R mutations (32, 38). Nonetheless, despite having these mutations, the binding ability of Omicron to the ACE2 receptor is weaker than the Alpha and Delta variants; this is explained by the presence of other mutations, such as K417N and E484A, which can cause a significant loss of polar interactions between the variant and the receptor and offset the enhanced interactions built by other mutations (39, 40). Additionally, the R493Q mutation, unique to the Omicron variant, has also been proven to have suboptimal binding to the ACE2 receptor, as its reversal increases its affinity for ACE2 (41).

Not only do specific mutations change the virus transmission capacity but the mutations can also impact the clinical progression and the overall clinical manifestation, such as symptoms (42). In this sense, the Delta VOC (that caused new infections waves in India) has 12 mutations in the RBD of the S protein, from which T478K, P681R, and L452R have been associated with this variant’s increased infectiousness and immune evasion capacity (43, 44) and could be involved in differences in the immune response against this variant and, therefore, with differences in the symptomatology. On the other hand, the Omicron variant carries more than 30 mutations, of which 15 are present in the RBD (32). Altogether, all mutations in the variants could account for the differences that we and others have found in symptoms among the patients (45), where fever, dry cough, headache, and fatigue were the principal reported symptoms in Delta-infected patients, whereas Omicron-infected cases were better characterized by having a sore throat; WT cases were characterized by those of the Delta cases plus anosmia and dysgeusia. However, to date, mutations assessed by the molecular SARS-CoV-2 VOC screening test have not shown a clinical implication via an immune response augmentation; therefore, further evaluation in this field is critical for better comprehending the clinical behavior of these variants.

We observed that asymptomatic cases predominated in the Omicron-infected group compared with the Delta and WT groups, which could contribute to increasing difficulty in detecting Omicron using a symptom-based testing approach. This finding aligns with other reports (46, 47), which could be explained because the Omicron variant tends to infect the upper respiratory tract rather than the lungs. Therefore, it has a reduced capacity to form syncytia in tissue culture (39, 46), and endosomal entry is its preferred route of infection (39), all of which have been associated with lower disease severity. Under the supposed more significant Omicron tropism in the upper respiratory tract, we observed that patients infected with this variant presented more rhinorrhea and sore throat than those infected with the other analyzed variants, similarly to that reported by Menni et al. (48).

Another important finding was that patients infected with the Omicron variant reported a lower loss of smell and taste prevalence than the WT or Delta variant, which was previously reported (48, 49). Another study found that 13–16% of patients lost their sense of smell and taste during the higher prevalence of the Omicron variant, while 44% of patients reported these symptoms when the Delta variant dominated (47). Therefore, the differential recovery rates among patients infected with different variants could be explained by the underlying mechanisms of tropism relative to different cells or respiratory tracts (50). Despite our findings, we do not rule out that the lower number of symptoms observed in patients infected with the Omicron variant is due in part to the fact that this group had fewer comorbidities than the other groups, as others have shown that presenting one or more comorbidities can result in an increased case fatality rate (51, 52).

Besides transmissibility and disease severity, neutralizing antibody evasion has also been reported to be VOC-dependent (53). Therefore, we conducted a follow-up of the patients via a telephone survey to determine whether they presented reinfection after vaccination. We found that all reinfections were associated with the Omicron variant, disregarding the vaccine platform used, which further supports the previous reports of an increased risk of reinfection with the emergence of Omicron as well as the molecular analysis that demonstrates the variant’s capacity to escape antibodies (11, 54).

The mutations that have been demonstrated to mediate escape from vaccine-induced neutralization are K417, E484, and N501 (39, 54) found in Omicron, Beta (B.1.351), and Gamma (P.1), respectively; however, studies have shown that Beta and Delta variants rarely cause reinfection (54), with Omicron being the VOC most associated with patients who experience reinfection. The advantage of the Omicron variant relies on the high number of mutations that increase its transmissibility and immune evasion capability. Even though the effects of most remaining Omicron mutations are still unknown (55), there is still much to know about the binding ability of Omicron to ACE2.

In conclusion, we show that the Omicron variant caused the biggest outbreak in Jalisco during the pandemic from late December 2021 to mid-February 2022 but with a less severe form than the one demonstrated by Delta and WT. The co-analysis of mutations and the clinical outcome is a public health strategy with the potential to infer mutations or variants that could increase disease severity and even indicate the long-term sequelae of COVID-19. We observed a notorious difference in symptoms preponderance among the VOC; asymptomatic and cases with sore throat characterize Omicron. Delta variant cases were distinguished by fever, dry cough, headache, and fatigue, while WT cases are similar to Delta plus anosmia and dysgeusia. Finally, Omicron VOC was also distinctive due to its reinfection capacity, which was 9.5% in previously vaccinated cases in our population. Moreover, our methodology displays the advantage of employing the SNP RT-PCR-based technique for the real-time detection of variants for molecular epidemiological surveillance and its impact on public health measures.

One of the main strengths of our study is its sample size and real-time assessment of the patient’s symptoms and the mutations associated with circulating genomic variants. On the other hand, among the limitations of our study, we state that we could not detect the duration of infection by the variants, and hospital admission was not ascertained in all patients. Moreover, some participants might omit some symptoms as these were self-reported, and we omit potential confounders such as drugs, which could modify some symptoms. In addition, we could not confirm the absence of previous infections in patients. Finally, for the reinfection analysis, although we considered the vaccination status (one or two doses), we could not match the time elapsed since vaccination.

## Data availability statement

The raw data supporting the conclusions of this article will be made available by the authors, without undue reservation.

## Ethics statement

The studies involving human participants were reviewed and approved by the Comité de Ética en Investigación (CEI-CUCS) Comité de Investigación (CI-CUCS) Comité de Bioseguridad (CBS-CUCS). The patients/participants provided their written informed consent to participate in this study.

## Author contributions

JH, OG, and JFM: conceptualization. HC, FR, OV, MG, MP, NV, and JCM: methodology. MP, OV, MG, and NV: formal analysis. MP, HC, OG, and JCM: data curation. MP, OV, and MG: writing—original draft preparation. JH, NV, and JFM: writing—review and editing, funding acquisition. All authors have read and agreed to the published version of the manuscript.

## Funding

This project was funded by the Universidad de Guadalajara (project nos. 260761 and 260998).

## Conflict of interest

The authors declare that the research was conducted in the absence of any commercial or financial relationships that could be construed as a potential conflict of interest.

## Publisher’s note

All claims expressed in this article are solely those of the authors and do not necessarily represent those of their affiliated organizations, or those of the publisher, the editors and the reviewers. Any product that may be evaluated in this article, or claim that may be made by its manufacturer, is not guaranteed or endorsed by the publisher.
